# Evolution of sperm morphology in anurans: insights into the roles of mating system and spawning location

**DOI:** 10.1186/1471-2148-14-104

**Published:** 2014-05-15

**Authors:** Yu Zeng, Shang Ling Lou, Wen Bo Liao, Robert Jehle

**Affiliations:** 1 Key Laboratory of Southwest China Wildlife Resources Conservation (Ministry of Education), China West Normal University, Nanchong 637009, P. R. China; 2 China Three Gorges Corporation, Beijing 100038, China; 3 School of Environment & Life Sciences, University of Salford, Salford M5 4WT, UK

**Keywords:** Anurans, Mating system, Spawning location, Sperm morphology, Sperm competition, Testes size

## Abstract

**Background:**

The degree of postcopulatory sexual selection, comprising variable degrees of sperm competition and cryptic female choice, is an important evolutionary force to influence sperm form and function. Here we investigated the effects of mating system and spawning location on the evolution of sperm morphology in 67 species of Chinese anurans. We also examined how relative testes size as an indicator of the level of sperm competition affected variation in sperm morphology across a subset of 29 species.

**Results:**

We found a significant association of mating system and spawning location with sperm morphology. However, when removing the effects of body mass or absolute testes mass for species for which such data were available, this effect became non-significant. Consistent with predictions from sperm competition theory, we found a positive correlation between sperm morphology and relative testes size after taking phylogeny into account.

**Conclusions:**

Our findings suggest that sexual selection in Chinese anurans favors longer sperm when the level of sperm competition is high. Pre-copulatory male-male competition and spawning location, on the other hand, do not affect the evolution of sperm morphology after taking body mass and absolute testes mass into account.

## Background

Spermatozoa exhibit a striking degree of variation in size and shape within and across species [1-3]. In addition to phylogeny and the mode of fertilization [4,5], postcopulatory sexual selection, comprising variation in the level of sperm competition [6] and cryptic female choice [7], is thought to be one of the main selective forces responsible for variation in sperm morphology [8-13]. However, the detailed evolutionary causes and consequences of the remarkable diversity of spermatozoa are still poorly understood.

Since the main biological role of sperm is to fertilize eggs, such variation is intimately associated with sperm function. Theoretical approaches to understand variation in sperm morphology are often based on assumptions concerning the relationship between sperm size and parameters such as sperm swimming speed and longevity [14,15]. These models predict that an increased risk of sperm competition among species can favor longer sperm with higher swimming speed. Empirical studies on a broad range of species including internal and external fertilizers have however resulted in empirical evidence which both supports [13,16-21] and rejects [22-25] associations between sperm size and swimming speed. It has further been suggested that the ratio between head length and tail length is a good predictor for the swimming speed of sperm [7,26,27]. Simpson et al. [27] reveal that sperm with a relatively long flagellum swam faster in external fertilizers, in which females have no opportunity to affect sperm motility. By contrast, sperm with a relatively short flagellum swam faster in internally fertilizing species where females can significantly affect sperm motility.

Sperm competition occurs when sperm from different males attempt to fertilize the same set of ova [6]. Males in polygamous species should suffer a higher risk of sperm competition than their monogamous counterparts. As a response, many studies showed that polygamous males have larger relative testes size [14,16,28-32] and longer sperm [9,11,16,33-37]. However, the association between sperm competition and sperm size could not be confirmed across all groups [38-41], and it has been suggested that that evolution of sperm morphology might also be influenced by the need to overcome hydrodynamic drag associated with the complexity of the sperm head [42]. A growing body of evidence suggests that species which experience greater levels of sperm competition have faster swimming sperm than species where sperm competition is relaxed or absent [16,20,22] but see also [13].

There is every reason to expect that the spawning environment should further affect sperm morphology [11,43,44]. For example, Byrne et al. [11] revealed for anurans that species with terrestrial spawning have a larger sperm head and a longer sperm tail than aquatically spawning species. However, more subtle effects of spawning environments on the evolution of sperm morphology across a broad range of anurans still await further exploration.

Here, we investigated the effects of mating system and spawning location on the evolution of sperm morphology in Chinese anurans. We also examined the amount of covariation between relative testes size (as an indicator of level of sperm competition) and sperm morphology. The aim of this study was to investigate patterns and possible causes of variation in sperm morphology in anurans, and to test the hypothesis that polygamous species have longer sperm than their monogamous counterparts. To this end, we analyzed a dataset on sperm morphology comprising 67 anurans from the Hengduan Mountains, China. We further analysed a data set on 29 species which also covered information on absolute testes mass and body mass, to test whether relative testes size is correlated with of the level of sperm competition and sperm morphology.

## Methods

The reported experiments comply with the current laws of China concerning animal experimentation, and permission to collect anurans was received from the Ethical Committee for Animal Experiments in China. All experiments involving the sacrifice of these live animals were approved by the Animal Ethics Committee at China West Normal University. All fieldworks performed were complied with the Convention on Biological Diversity and the Convention on the Trade in Endangered Species of Wild Fauna and Flora local ethical regulations and agreements.

For our analyses we combined our primary data with data derived from the literature; taken together, we compiled a data set on sperm morphology for 67 species, and a further data set which also incorporates body mass and absolute testes mass for 29 species (Additional file 1: Table S1). To obtain the primary data, we collected 5–6 males of each species by hand at night using a flashlight during the breeding season at the Hengduan Mountains, China, from 2009 to 2013. Individuals were kept singly in wire-netting rectangular containers (20 × 10 × 15 cm; L × W × H) placed in a tank (90 × 40 × 40 cm; L × W × H) with a depth of 10 cm of fresh water at room temperature. We weighed body mass to the nearest 0.1 mg with an electronic balance and killed animals by double-pithing. We dissected all individuals and removed both testes. After weighing the testes to the nearest 0.1 mg, we immediately crushed them and released sperm into reverse-filtered tap water. We pipetted 50 μl of the suspension onto microscope slides and air-dried the slides before staining them with acid carmine for 40 seconds. We then captured images of mature sperm using a Motic BA300 digital camera attached to a Moticam2006 light microscope at a 400× magnification. Abnormal spermatozoa (broken tail, damaged or missing acrosome) were not considered in the analysis. Sperm morphology (total length, head length and tail length) was measured using a line chain tool in the Motic Images Advanced 3.2 software. Measurements comprised 20 sperms from each male. All measurements (testes and sperm) were taken without knowledge of the species identification to prevent observer bias. The repeatability [45] was high when we compared three measurements on 20 sperm (R = 0.94). To further enhance the reliability of sperm size data, we measured the same 20 spermatozoa three times, using average values in the analysis. For the three species *Rhacophorus chenfui*, *R. dugritei* and *R. omeimontis*, the length of sperm heads was calculated as L = πDN (L: length of sperm head, D = diameter of helix, N = number of turns in the helix) [46]. We used the ratio between flagellum and head length as a possible predictor of sperm swimming speed [26].

Following Byrne and Roberts [47], we used mating system as an imperfect surrogate for the intensity of sperm competition on a two-point scale: 1 = simultaneous polyandry, where multiple males clasp a female and sperm from males to simultaneously compete to fertilize eggs over the course of a breeding season; 2 = monandry, where a female mates with one male over the course of a breeding season and deposits a single clutch. In *Chaparana quadrana* we observed that multiple males participated in fertilising the eggs deposited by a single female without amplexus (similar to *C. taihangnicus*) [48], and we regarded this species as simultaneously polyandrous. Following Li et al. [49], we classified spawning location on a four-point scale: 1 - arboreal: spawning occurs mostly occur on trees, eggs in foam nests; 2 - terrestrial: spawning occurs on the ground, eggs laid in foam nests in holes or on the ground near ponds; 3 - lentic aquatic – eggs in ponds; 4 - lotic aquatic – eggs in running water.

Comparative analyses of interspecific data may require phylogenetic control, as closely related species share parts of their evolutionary history. To control for phylogeny we employed comparative analyses by independent contrasts [50]. We used an established phylogeny [51,52] to reconstruct phylogenetic trees for the 67 and 29 anurans species, respectively (Additional file 2: Figure S1 and Additional file 3: Figure S2). Because information on branch lengths was not available, they were first arbitrarily set to 1 based on the suggestions of Pagel [53]. Felsenstein [54] provided the details of the general procedure for estimating the character values in the ancestors. With 67 and 29 species at the tips of the reconstructed trees, respectively, 66 (67–1) and 28 (29–1) pairs of contrasts could be computed for pairs of nodes sharing an immediate common ancestor, and then re-scaled and analysed as suggested by Garland et al. [55].

Allometric effects were controlled for by correcting for body mass. All data were log-transformed in all analyses. None of the distributions of log-transformed variables (such as body mass, absolute testes mass or sperm morphology) were significantly different from normal, and we used parametric tests throughout.

In the two datasets used in the GLMs, there was only a small number of polygamous, arboreal and terrestrial species compared to larger numbers of monandrous species and the two aquatic categories. This made it difficult to compare the effects of mating system and spawning location per se with an analysis which also takes the effects of absolute testes mass and body mass into account. Hence, we combined arboreal and terrestrial species. Both are characterized by foam nests, which as a hypothesis enable fertilization in a rather protected environment. As a result, the categories of spawning locations as terrestrial, lentic and lotic were used in all analysis.

To test for the effect of mating system and spawning locations on sperm morphology among 67 species, we first used a multivariate GLM to test for the effects of mating system and spawning locations on independent contrasts in sperm morphology, using independent contrasts in sperm morphology as dependent variables and mating system and spawning locations as fixed factors. For the 29 species for which the required data were available, we conducted a multivariate GLM on independent contrasts in testis mass as dependent variables and mating system and spawning location as fixed factors and independent contrasts in body mass as covariate to test relative testes size differences. In order to control for collinearity between mating system and relative testes mass, we also ran a separate analysis with relative testes mass, spawning location and body mass; obtaining the same results for both analyses would lend support to an association between them. We also ran GLMs with sperm morphology as dependent variable, mating system/spawning location, species types and their interaction as fixed factors to test the difference in relationship between sperm morphology and mating system/spawning location between 67 and 29 species. Finally, we used phylogenetically controlled multiple regression models (i.e., including body mass as a covariate) to test for correlations between independent contrasts in relative testes size and sperm morphology. All tests were conducted by using Type III sums of squares.

## Results

**Figure 1:**
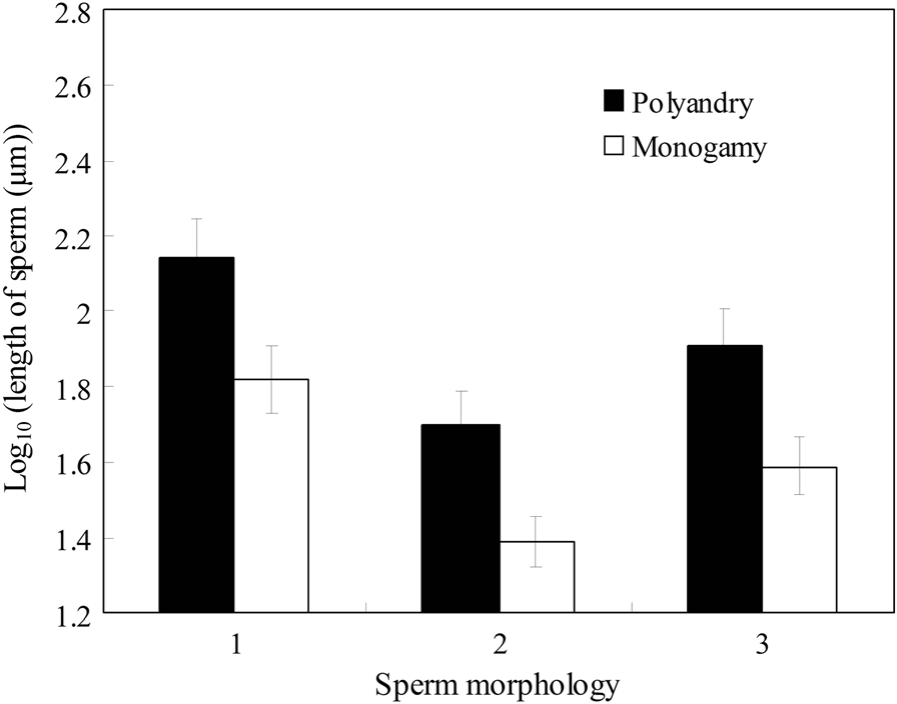
**Differences in sperm morphology between polyandrous and monogamous mating system across 67 anurans species.** 1 – sperm total length; 2 – sperm head length; 3 – sperm tail length.

**Figure 2:**
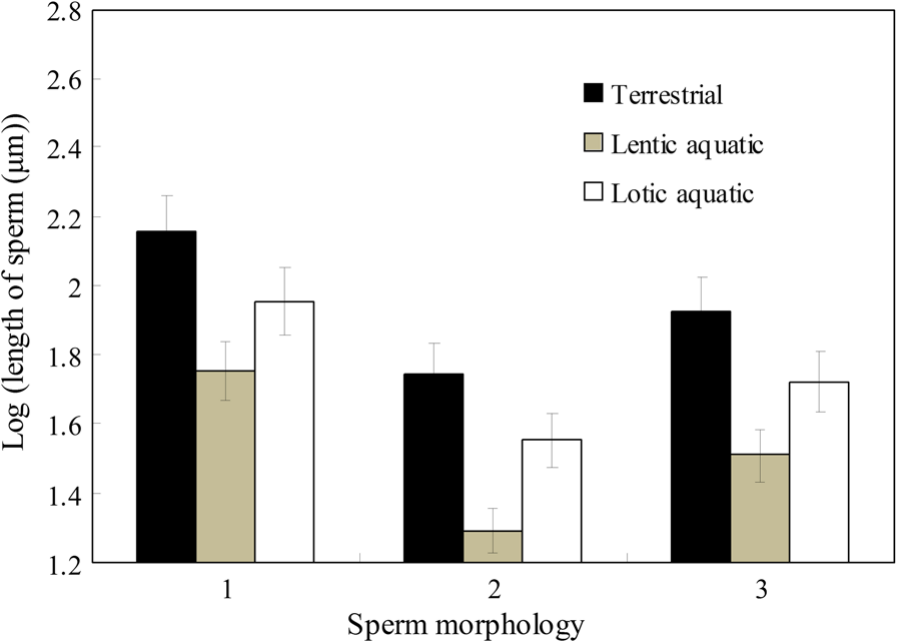
**Differences in sperm morphology among spawning sites across 67 anurans species.** 1 – sperm total length; 2 – sperm head length; 3 – sperm tail length.

We used GLMs to determine if sperm morphology is influenced by the mating system and spawning location in 67 anurans. Mating system and spawning location significantly affected independent contrasts in total sperm length, head length and tail length, but not the ratio between sperm head length and tail length (Table 1). Polyandrous species had longer sperm than monogamous species (Figure 1). Furthermore, post-hoc tests revealed that there was no difference in tail length of sperm between species with terrestrial and lotic oviposition (*P* = 0.058). However, species with terrestrial oviposition had longer sperm than those with aquatic oviposition (all *P* < 0.045). Species with lotic oviposition had longer sperm than those with lentic oviposition (Figure 2; both *P* < 0.008).

**Table 1:** **The influences of mating system and spawning locations on variation in independent contrasts in sperm morphology across 67 anurans species using GLM**^**a**^

**Source**	**Sums of squares**	**d.f.**	**Mean square**	***F***	***P***
Sperm total length					
Mating system	0.034	1	0.034	4.864	0.031
Spawning locations	0.059	2	0.029	4.421	0.016
Sperm head length					
Mating system	0.086	1	0.086	9.016	0.004
Spawning locations	0.120	2	0.060	6.514	0.003
Sperm tail length					
Mating system	0.057	1	0.057	5.401	0.023
Spawning locations	0.085	2	0.043	4.132	0.021
Ratio of head to tail					
Mating system	0.441	1	0.441	0.158	0.694
Spawning locations	56.556	2	28.278	0.667	0.517

^a^For testing evolutionary associations, the regression was forced through the origin.

Species with a polygamous mating system had significantly larger relative testes mass than monogamous species when correcting body mass (phylogenetically controlled GLMs, *F*_1, 27_ = 9.936, *P* < 0.001). Relative testes mass also significantly differed among spawning locations (phylogenetically controlled GLMs, *F*_2, 27_ = 3.661, *P* = 0.041).

The mating system and spawning location significantly affected sperm morphology across the 29 species for which data on absolute testes mass were available (phylogenetically controlled GLMs: mating system, *F*_1, 27_ > 4.343, *P* < 0.038; spawning location, *F*_2, 27_ > 3.953, *P* < 0.029). Neither mating system nor spawning location affected the ratio between head length and tail length (mating system: *F*_1, 27_ = 1.201, *P* = 0.283; spawning location: *F*_2, 27_ = 0.950, *P* = 0.400). However, when this analysis is based on mating system and spawning location as two predictors, the independent contrasts in sperm morphology did not differ between polygamous and monogamous mating systems and spawning locations (Table 2). We also found that independent contrasts in sperm morphology were unrelated to spawning location (*F*_2, 27_ < 2.817, *P* > 0.085) and relative testes mass (*F*_1, 27_ < 2.115, *P* > 0.159) when mating system was replaced by relative testes mass in the model. The converging results for both analyses demonstrate the lack of collinearity between mating system and relative testes size.

**Table 2:** **The influences of mating system and spawning locations on variation in independent contrasts in sperm morphology across 29 anurans species when correcting the body mass using GLM**^**a**^

**Source**	**Sums of squares**	**d.f.**	**Mean square**	***F***	***P***
Sperm total length					
Mating system	0.001	1	0.001	0.007	0.932
Spawning locations	0.031	2	0.016	2.266	0.127
Body mass	0.122	1	0.122	17.655	<0.001
Sperm head length					
Mating system	0.006	1	0.006	0.500	0.487
Spawning locations	0.019	2	0.009	0.785	0.468
Body mass	0.158	1	0.158	13.365	0.001
Sperm tail length					
Mating system	0.002	1	0.002	0.199	0.660
Spawning locations	0.059	2	0.030	2.849	0.079
Body mass	0.206	1	0.206	19.880	<0.001
Ratio of head to tail					
Mating system	0.060	1	0.060	0.027	0.870
Spawning locations	0.451	2	0.225	0.103	0.903
Body mass	0.375	1	0.375	0.171	0.684

^a^For testing evolutionary associations, the regression was forced through the origin.

We found significant differences in mating systems between spawning locations when considering 67 species (*F*_2,66_ = 35.303, *P* < 0.001) as well as when focusing on the 29 species for which data on absolute testes mass were available (*F*_2,28_ = 13.152, *P* < 0.001). The GLMs also revealed non-significant differences in the relationship between sperm morphology and mating system as well as spawning location between both datasets (Table 3), suggesting that body mass and absolute testes mass rather than species number affect variation in sperm morphology.

**Table 3:** **Differences in relationship between sperm morphology and mating system/spawning location between 67 and 29 species using GLM**^**a**^

**Source**	**d.f.**	**Mean square**	***F***	***P***
Sperm total length				
Mating system	1	0.011	1.322	0.253
Spawning locations	2	0.017	2.212	0.126
Species types	1	0.006	0.663	0.418
Mating system* species types	1	0.015	1.751	0.189
Spawning locations* species types	2	0.014	1.664	0.230
Sperm head length				
Mating system	1	0.037	3.194	0.077
Spawning locations	2	0.023	2.037	0.137
Species types	1	0.032	2.799	0.098
Mating system* species types	1	0.030	2.578	0.112
Spawning locations* species types	2	0.013	1.163	0.317
Sperm tail length				
Mating system	1	0.011	0.796	0.375
Spawning locations	2	0.043	2.187	0.059
Types of species	1	0.004	0.264	0.608
Mating system* species types	1	0.037	2.700	0.104
Spawning locations* species types	2	0.044	3.155	0.054

^a^For testing evolutionary associations, the regression was forced through the origin.

*For interaction between two variables.

**Figure 3:**
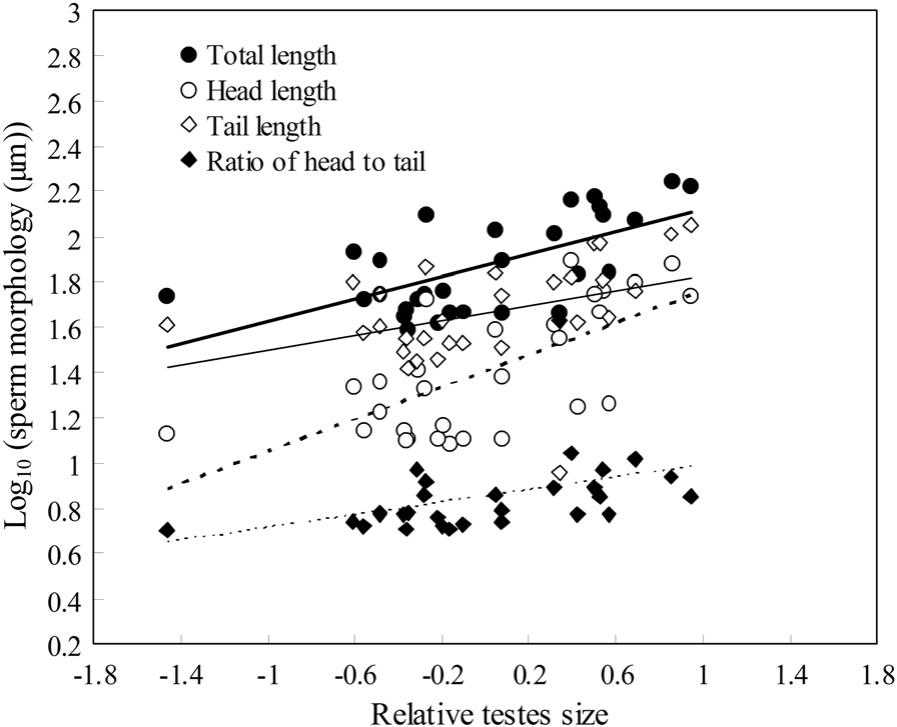
**Correlations between sperm morphology and relative testes size among 29 anurans species.** Relative testes size was from residuals of observed testes mass minus predicted testes mass on the basis of the allometric regression.

We further examined the correlation between relative testes mass and sperm morphology using phylogenetically controlled multiple regression models including body mass as a covariate. We found that independent contrasts in relative testes size were further positively correlated with independent contrasts in sperm morphology (total length, *t* = 3.229, *P* = 0.004; head length, *t* = 2.895, *P* = 0.008; tail length, *t* = 3.682, *P* = 0.005; ratio of head to tail, *t* = 2.465, *P* = 0.012; Figure 3).

## Discussion

Mating system and spawning location significantly affect sperm morphology among 67 anurans species. Polyandrous species have longer sperm than monogamous species. Species with arboreal spawning locations have longer sperm than species with aquatic spawning, and species with both terrestrial and lotic spawning locations have longer sperms than those with lentic spawning sites. These patterns remain unchanged when analyzing 29 species for which data on body mass and absolute testes mass are available. However, after correcting for the effects of body mass or absolute testes mass, the effects of mating system and spawning location on sperm morphology disappear. Moreover, we find that relative testes size (as a proxy for the risk of sperm competition) is positively correlated with sperm total length, head length and tail length. This finding is consistent with the prediction that sperm competition should favor longer sperm. A positive correlation between relative testes size and the ratio between sperm head size and tail size also suggests that sperm competition might promote faster sperm in anurans.

We find that sperm length varies by a factor of 8.1 across 67 Chinese anurans species. *Rhacophorus dennysi* have the longest sperm (235 μm), whereas *Hoplobatrachus tigrina* have the shortest sperm (29 μm)*.* An earlier study on myobatrachid frogs has suggested that variation in sperm head and tail length is not associated with variation in body size after controlling for phylogeny [11]. However, we observe a consistent positive correlation between sperm length and relative testes size. For Australian frogs, a number of selective pressures result in the observed variation in sperm morphology [11]. Our results provide evidence that particularly the mating system may account for the evolution in sperm morphology across Chinese anurans.

Across vertebrates, there is ample empirical evidence which supports predictions from sperm competition theory about sperm morphology at the interspecific level [8-12,37,43,56-59]. The majority of studies suggest that selection favors longer sperm when the intensity and risk of sperm competition is high. However, in a few cases, either negative relationships or no influence of sperm competition on sperm length are observed [39,40,42]. In our study, we provide clear evidence that more intense sperm competition results in longer sperm. Selection on relative testis size does not necessarily result in variation in sperm morphology. Sperm competition might result in selection for longer sperm if these have a competitive advantage and in selection for larger testes either because the production of longer sperm requires larger testes or because larger testes can produce more sperm (or both combined) [9,11,12,43]. The relationship between sperm morphology and relative testes mass was also supported by our finding that polygamous species have longer sperm than monogamous species.

The mating system is often used as an indicator of the intensity of sexual selection [60]. Our results show that sperm morphology is affected by mating system across 67 species, suggesting that more intense sexual selection results in longer sperm, in line with previous evidence from e.g. fishes [43]. Variation in sperm morphology could also be attributed to the intensity of sperm competition as measured by relative testis size. Furthermore, our study confirms previous studies for anurans which show that the mating system affects relative testes size [30,61-63].

The ratio between head length and tail length is frequently used as an indicator of sperm speed [17,26,27]. In our study across 67 species, the mating system does not affect this ratio, suggesting that the intensity of male-male competition does not increase putative sperm swimming speed. However, a positive correlation between relative testes size and the ratio between head and tail length across the second data set (29 species) reveals that sperm competition can promote sperm with short tails and long head, implying that sperm swimming speed is slower when the risk of sperm competition is high. In line with results from other studies [2,64-66] this suggests that there is a possible interaction between sperm longevity and swimming speed. Based on theory, an increase of sperm competition should favor increasing or decreasing sperm length when sperm longevity is negatively or positively correlated with sperm length, respectively [15]. It is important to consider that differences in sperm morphology, velocity and longevity between anurans and fish might arise from different targets of selection. Assuming a trade-off between sperm velocity and viability, selection can act on sperm to be fast (and hence short-lived due to energy depletion) or to live longer (and thus being slower as a consequence). An important difference between fish and anurans, for example, is that anuran sperm often have to penetrate layers of jelly and so can stay motile for over an hour, although swimming slowly, to work their way through. In most externally fertilizing fish, however, sperm tend to be released close to the eggs and so swim a short distance through water, where speed might be more important than longevity. This may then also be reflected in differences in sperm morphology and selection on it. In addition, there are likely to be differences in the risk of sperm loss or dilution, so that any trade-off between sperm size and number might also vary between these taxa and differentially constrain the evolution of sperm morphology. As a consequence, it depends on the interpretation of the theoretical predictions as to how to interpret Stockley et al.’s results. Contrary to this prediction, however, sperm length in fish is significant negatively correlated with sperm competition despite a negative correlation between sperm longevity and sperm length [39]. Unfortunately we currently lack data on the relationship between sperm length and longevity for anurans.

Amphibians deposit their eggs on a range of aquatic and terrestrial substrates [49,67,68], and previous studies have shown that sperm morphology can depend on spawning location [11,69]. Byrne et al. [11] reveal that spawning location significantly affects the length of sperm heads, but not the length of sperm tails. In our study, terrestrial species have longer sperm than aquatic species, and the shortest sperm is found in species with lentic aquatic ovipositing. In line with a previous study on fish which showed that buccal-fertilizing cichlids having shorter sperm than substrate fertilizers [43], this suggests that the locomotor ability of sperm needs to be higher in lotic sites than in lentic sites. However, when correcting for the effects of body mass and absolute testes mass, these relationships disappear, and our data therefore do not provide clear evidence for a link between water turbulence affecting sperm total length across 29 species.

## Conclusion

In conclusion, our study from data on sperm morphology shows that mating system and spawning location significantly influence the evolution of sperm morphology, but that the effect is largely due to differential body mass and absolute testes mass [70]. We find that the influence of the mating system and spawning location on the evolution of sperm morphology remains unchanged when accounting for phylogeny. We also find a relationship between sperm morphology and relative testes size, suggesting that the risk of sperm competition has a strong influence on sperm morphology.

## Availability of supporting data

The sperm morphology data set supporting the results of this article is available in the Dryad Digital Repository, with identifier doi:10.5061/dryad.30951 (http://datadryad.org/resource/doi:10.5061/dryad.30951).

## Competing interests

The authors have declared that no competing interests exist.

## Authors’ contributions

YZ and SLL carried out the analyses and drafted the manuscript. WBL and YZ designed the study. RJ helped finalising the manuscript. All the authors read and approved the final manuscript.

## Supplementary Material

Additional file 1: Table S1 Species, mean body mass, absolute testes mass, sperm morphology, mating system, ovipostion locations and references of published papers. For spawning location we placed species into one of four nominal categories, 1 - arboreal; 2 - terrestrial; 3 - lentic aquatic. 4 - lotic aquatic; Mating system as an imperfect surrogate of the intensity of sexual selection was quantified on a two-point scale: 1 - simultaneous polyandry where sperm from multiple males compete to fertilize eggs of a female over the course of a breeding season; 2 monandry where a females mates with one male over the course of a breeding season by depositing a single clutch (following Byrne et al. [11]). Click here for file

Additional file 2: Figure S1 The phylogenetic tree of the 67 anurans species used in the comparative analysis following Jiang et al. [51] and Pyron and Wiens [52]. Click here for file

Additional file 3: Figure S2 The phylogenetic tree of the 29 anurans species used in the comparative analysis following Jiang et al. [51] and Pyron and Wiens [52]. Click here for file
