# Supplementary material for: Evolution of sperm morphology in anurans: insights into the roles of mating system and spawning location
Source: BMC Evol Biol. 2014 May 15;14:104. doi: 10.1186/1471-2148-14-104 (PMC4030069; doi:10.1186/1471-2148-14-104)
Supplement: Additional file 1: Table S1 — Species, mean body mass, absolute testes mass, sperm morphology, mating system, ovipostion locations and references of published papers. For spawning location we placed species into one of four nominal categories, 1 - arboreal; 2 - terrestrial; 3 - lentic aquatic. 4 - lotic aquatic; Mating system as an imperfect surrogate of the intensity of sexual selection was quantified on a two-point scale: 1 - simultaneous polyandry where sperm from multiple males compete to fertilize eggs of a female over the course of a breeding season; 2 monandry where a females mates with one male over the course of a breeding season by depositing a single clutch (following Byrne et al. [11]). [file 1471-2148-14-104-S1.doc]

Additional file 1: Table S1. Species, mean body mass, testes mass, sperm morphology, mating system, ovipostion locations and references of published papers. For spawn location we placed species into one of four nominal categories, 1 = Arboreal; 2 = Terrestrial; 3 = Lentic aquatic. 4 = Lotic aquatic; Mating system as an imperfect surrogate of the intensity of sexual selection on a two-point scale: 1 = Simultaneous polyandry where sperm from multiple males compete to fertilize eggs of a female over the course of a breeding season; 2 = Monandry where a females mates with one male over the course of a breeding season by depositing a single clutch.

| Species | Body mass  (g) | Testes mass  (mg) | Total length  (μm) | Head length  (μm) | Flagellum length (μm) | Mating system | Oviposition habitat | References |
| --- | --- | --- | --- | --- | --- | --- | --- | --- |
| *Rhacophorus chenfui* | 6.92 | 131.3 | 121.4 | 63.2 | 58.2 | 1 | 2 | Own data |
| *Rhacophorus dugritei* | 7.07 | 94.6 | 125.2 | 58.2 | 65.0 | 1 | 2 | Own data |
| *Rhacophorus omeimontis* | 15.80 | 446.6 | 178.8 | 75.4 | 103.4 | 1 | 1 | Own data |
| *Rhacophorus dennysi* |  |  | 235.0 | 76.0 | 159.0 | 1 | 1 | 1,8,9 |
| *Polypedates megacephalus* | 8.24 | 101.1 | 150.2 | 55.8 | 94.4 | 1 | 2 | Own data |
| *Polypedates mutus* |  |  | 228.8 | 95.3 | 133.5 | 1 | 2 | 1,8,9 |
| *Branchytarsophrys feae* |  |  | 156.7 | 82.4 | 74.3 | 1 | 4 | 4,6,8 |
| *Branchytarsophry*s *chuannanensis* | 100.82 | 1048 | 144.9 | 79.5 | 65.4 | 1 | 4 | Own data |
| *Ophryophryne microstoma* |  |  | 94.8 | 44.9 | 49.9 | 2 | 4 | 4,6,8 |
| *Megophrys minor brachykolos* |  |  | 118.6 | 56.5 | 62.1 | 2 | 4 | 4,6,8 |
| *Megophrys minor binchuanensis* |  |  | 135.2 | 64.1 | 71.1 | 2 | 4 | 4,6,8 |
| *Megophrys shapingensis* |  |  | 186.7 | 107.3 | 79.4 | 2 | 4 | 4,6,8 |
| *Megophrys omeimontis* |  |  | 126.5 | 57.6 | 68.9 | 2 | 4 | 4,6,8 |
| *Megophrys glandulosa* |  |  | 102.6 | 41.2 | 61.4 | 2 | 4 | 4,6,8 |
| *Megophrys boettgeri* |  |  | 105.9 | 46.2 | 59.7 | 2 | 4 | 4,6,8 |
| *Bufo gargarizans* | 34.56 | 34.6 | 84.6 | 22.1 | 63.5 | 2 | 3 | Own data |
| *Bufo andrewsi* | 46.37 | 61.7 | 79.1 | 23.0 | 56.1 | 2 | 3 | Own data |
| *Bufo minshanicus* |  |  | 71.9 | 18.6 | 46.1 | 2 | 3 | Own data |
| *Bufo tuberculatus* |  |  | 75.3 | 22.6 | 47.1 | 2 | 3 | 5,8,9 |
| *Bufo tibetanus* | 20.30 | 95.5 | 79.6 | 24.1 | 55.5 | 2 | 3 | Own data |
| *Bufo melanosctictus* |  |  | 65.3 | 18.8 | 40.2 | 2 | 3 | 5,8,9 |
| *Bufo raddei* |  |  | 62.0 | 23.0 | 49.0 | 2 | 3 | 5,8 |
| *Pelophylax hubeinesis* |  |  | 44.7 | 15.5 | 29.2 | 2 | 3 | 2,8 |
| *Pelophylax plancyi* |  |  | 51.0 | 13.0 | 38.0 | 2 | 3 | 2,8 |
| *Pelophylax pleuraden* | 9.59 | 25.8 | 45.7 | 11.9 | 33.7 | 2 | 3 | Own data |
| *Pelophylax nigromaculata* | 22.03 | 24.1 | 52.2 | 13.9 | 38.3 | 2 | 3 | Own data |
| *Hylarana guentheri* | 22.30 | 39.0 | 39.3 | 12.8 | 26.5 | 2 | 3 | Own data |
| *Fejervarya limnocharis* | 4.56 | 20.6 | 45.6 | 13.0 | 32.6 | 2 | 3 | Own data |
| *Microhyla ornata* | 0.72 | 1.1 | 44.8 | 13.7 | 31.1 | 2 | 3 | Own data |
| *Microhyla mixturi* |  |  | 51.0 | 15.2 | 45.2 | 2 | 3 | 2,8 |
| *Microhyla heymonri* |  |  | 41.2 | 13.0 | 28.2 | 2 | 3 | 2,8 |
| *Amolops lifanensis* | 14.35 | 44.8 | 46.7 | 12.8 | 33.9 | 2 | 4 | Own data |
| *Amolops mantzorum* | 17.36 | 22.6 | 56.2 | 16.8 | 39.4 | 2 | 4 | Own data |
| *Amolops wuyiensis* |  |  | 68.0 | 16.2 | 51.8 | 2 | 4 | 2,8,9 |
| *Hyla annectans chuanxiensis* | 3.50 | 49.1 | 71.5 | 18.1 | 43.4 | 1 | 3 | Own data |
| *Hyla annectans jingdongensis* | 3.17 | 31.8 | 69.5 | 17.9 | 41.6 | 1 | 3 | Own data |
| *Hyla arborea* |  |  | 46.7 | 15.0 | 31.7 | 2 | 3 | 2,8 |
| *Hyla sanchiangensis* |  |  | 56.0 | 18.5 | 37.5 | 2 | 3 | 2,8,9 |
| *Hyla tsinlingensis* |  |  | 56.6 | 15.5 | 41.1 | 2 | 3 | 2,8 |
| *Kaloula verrucosa* | 5.49 | 12.7 | 41.9 | 13.0 | 28.9 | 2 | 3 | Own data |
| *Kaloula borealis* |  |  | 60.3 | 12.8 | 47.5 | 2 | 3 | 2,8 |
| *Odorrana livida* |  |  | 52.0 | 22.0 | 32.0 | 2 | 4 | 2,8 |
| *Odorrana grahami* | 22.93 | 47.9 | 56.6 | 21.5 | 35.1 | 2 | 4 | Own data |
| *Odorrana margaretae* | 33.19 | 64.9 | 54.1 | 25.6 | 28.5 | 2 | 4 | Own data |
| *Odorrana tormotus* |  |  | 77.5 | 26.0 | 51.5 | 2 | 4 | 2,8 |
| *Rana omeimontis* | 11.87 | 1.6 | 54.4 | 13.6 | 40.8 | 2 | 3 | Own data |
| *Rana chensinensis* | 9.94 | 24.7 | 57.6 | 14.7 | 42.9 | 2 | 3 | Own data |
| *Rana chaochiaoensis* | 8.18 | 13.8 | 47.4 | 12.6 | 35.8 | 2 | 3 | Own data |
| *Rana japonica* |  |  | 52.0 | 17.0 | 35.0 | 2 | 3 | 2,8,9 |
| *Rana adenopleura* |  |  | 55.0 | 15.0 | 40.0 | 2 | 2 | 2,8,9 |
| *Rana tiantaiensis* |  |  | 47.8 | 17.8 | 30.0 | 2 | 3 | 2,8,9 |
| *Hoplobatrachus tigrina* |  |  | 29.0 | 13.9 | 15.1 | 2 | 4 | 2,8,9 |
| *Paa shini* |  |  | 72.6 | 28.3 | 44.3 | 2 | 4 | 7,8,9 |
| *Paa yunnanensis* | 30.02 | 409.3 | 138.9 | 46.4 | 92.5 | 2 | 4 | Own data |
| *Paa boulengeri* | 52.73 | 448.3 | 104.4 | 41.2 | 63.2 | 2 | 4 | Own data |
| *Paa robertingeri* |  |  | 84.8 | 30.4 | 54.4 | 2 | 4 | 7,8 |
| *Paa spinosa* |  |  | 91.3 | 30.3 | 61.0 | 2 | 4 | 7,8 |
| *Paa exilispinosa* |  |  | 103.4 | 40.2 | 63.2 | 2 | 4 | 7,8 |
| *Nanorana parkeri* |  |  | 118.7 | 55.8 | 59.2 | 2 | 3 | 7,8,9 |
| *Nanorana pleskei* | 17.53 | 37.3 | 126.3 | 52.2 | 74.1 | 2 | 3 | Own data |
| *Nanorana venbtripunctata* | 4.26 | 18.2 | 107.7 | 39.0 | 68.7 | 2 | 3 | Own data |
| *Chaparana quadrana* | 43.31 | 1556 | 167.8 | 54.7 | 113.1 | 1 | 4 | Own data |
| *Bombina maxima* | 18.96 | 166.3 | 45.4 | 36.2 | 9.2 | 2 | 3 | Own data |
| *Bombina orentalis* |  |  | 36.5 | 29.7 | 6.8 | 2 | 3 | 3,8 |
| *Bombina fortinuptialis* |  |  | 39.9 | 33.2 | 6.7 | 2 | 3 | 3,8 |
| *Bombina lichuanensis* |  |  | 41.0 | 33.2 | 6.8 | 2 | 3 | 3,8 |
| *Bombina microdeladigitora* |  |  | 40.8 | 33.4 | 7.4 | 2 | 3 | 3,8 |

**References**

1. Qin LM, Zheng ZH, Jiang JP, Xie F, Mo YM: **Sperm morphology of five *Rhacophorus* (Amphibia: Anura: Rhacophoridae) Species from China**. *Asian Herpetol Res* 2008, **11**: 105–109.

2. Liang RJ: **Morphologies of spermatozoa in 24 Chinese anurans species**. *Chin J Zool* 1994, **29**: 20–23.

3. Zheng ZH, Fei L, Ye CY: **Study on the morphology of spermatozoa of genus Bombina**. *Cultum Herpetol Sin* 2000, **8**: 222–227.

4. Zheng ZH, Fei L, Ye CY: **Study on morphology of spermatozoa of Megophrys from China**. *Chin J Appl Envir Biol* 2005, **6**: 161–165.

5. Li WY, Zheng ZH, Jiang JP, Xie F, Qin LM: **Sperm morphology of Bufo from China***. Chin J Zool* 2008, 43: 109–115.

6. Zheng ZH, Fei L, Ye CY, Xie F, Jiang JP: **Compare five study on sperm morphology of Chinese megophryinae and its taxonomical sense (Amphibia: Pelobaridae)**. *Acta Zoot Sin* 2002, 27: 167–172.

7. Qin LM: ***Study sperm morphology, ultrastructure and seasonal changes in testis of tribe Paini***. Chengdu: Ms. Degree; 2007.

8. Li C, Dai Q, Wang YZ, Gu HJ, Liu ZJ: **Reproductive modes in Sichuan Anurans**. *Biodiver Sci* 2005,**13**: 290–297.

9. Fei L, Hu SQ, Ye CY, Huang YZ: ***Fauna of sinica.*** [***Amphibia***](http://www.iciba.com/Amphibia)***, volume II, anuran***. Beijing: Science Press; 2009.
