# Supplementary figures and images for: Evolution of sperm morphology in anurans: insights into the roles of mating system and spawning location
Source: BMC Evol Biol. 2014 May 15;14:104. doi: 10.1186/1471-2148-14-104 (PMC4030069; doi:10.1186/1471-2148-14-104)

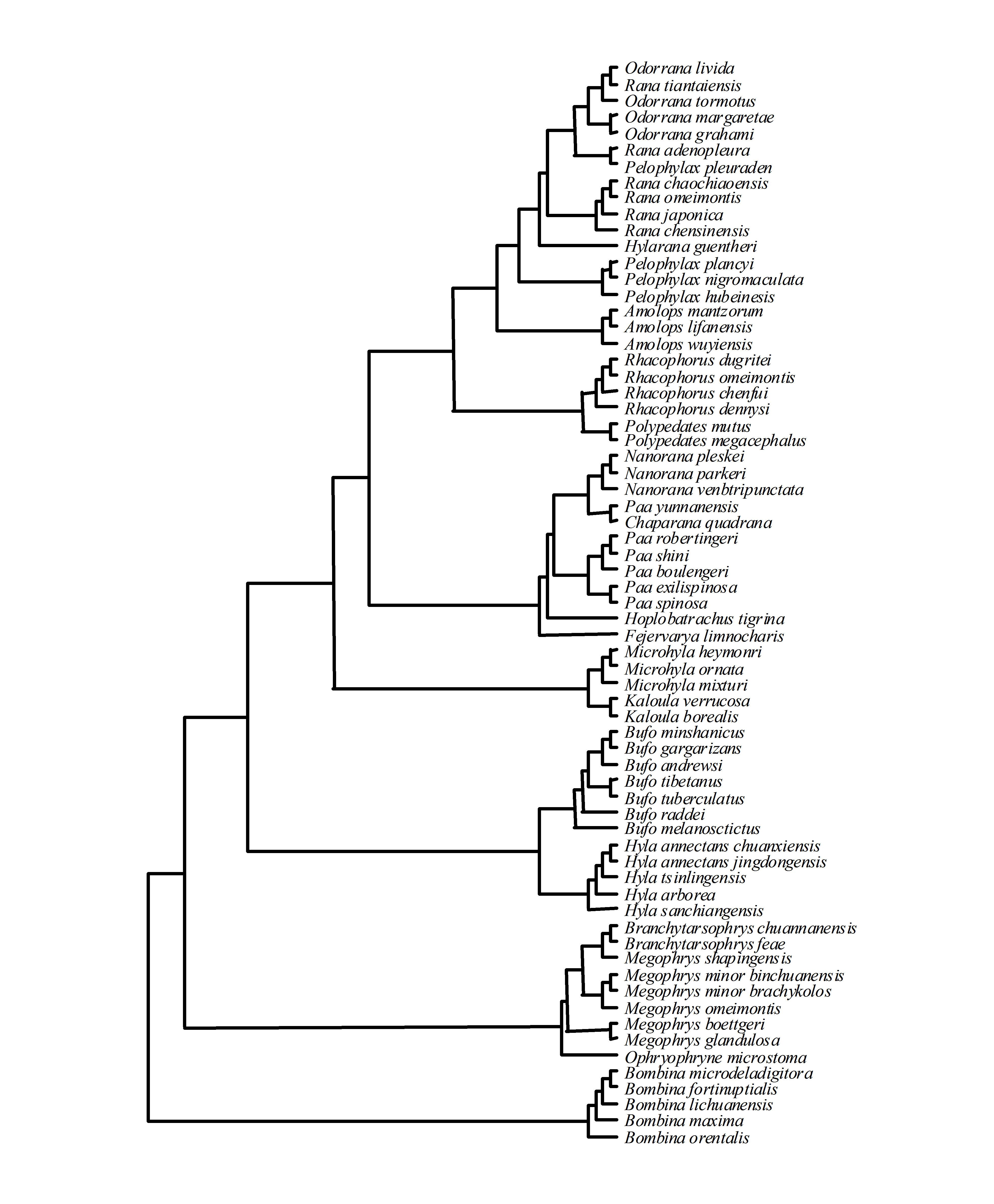

Supplement: Additional file 2: Figure S1 — The phylogenetic tree of the 67 anurans species used in the comparative analysis following Jiang et al. [51] and Pyron and Wiens [52]. [file 1471-2148-14-104-S2.jpeg]

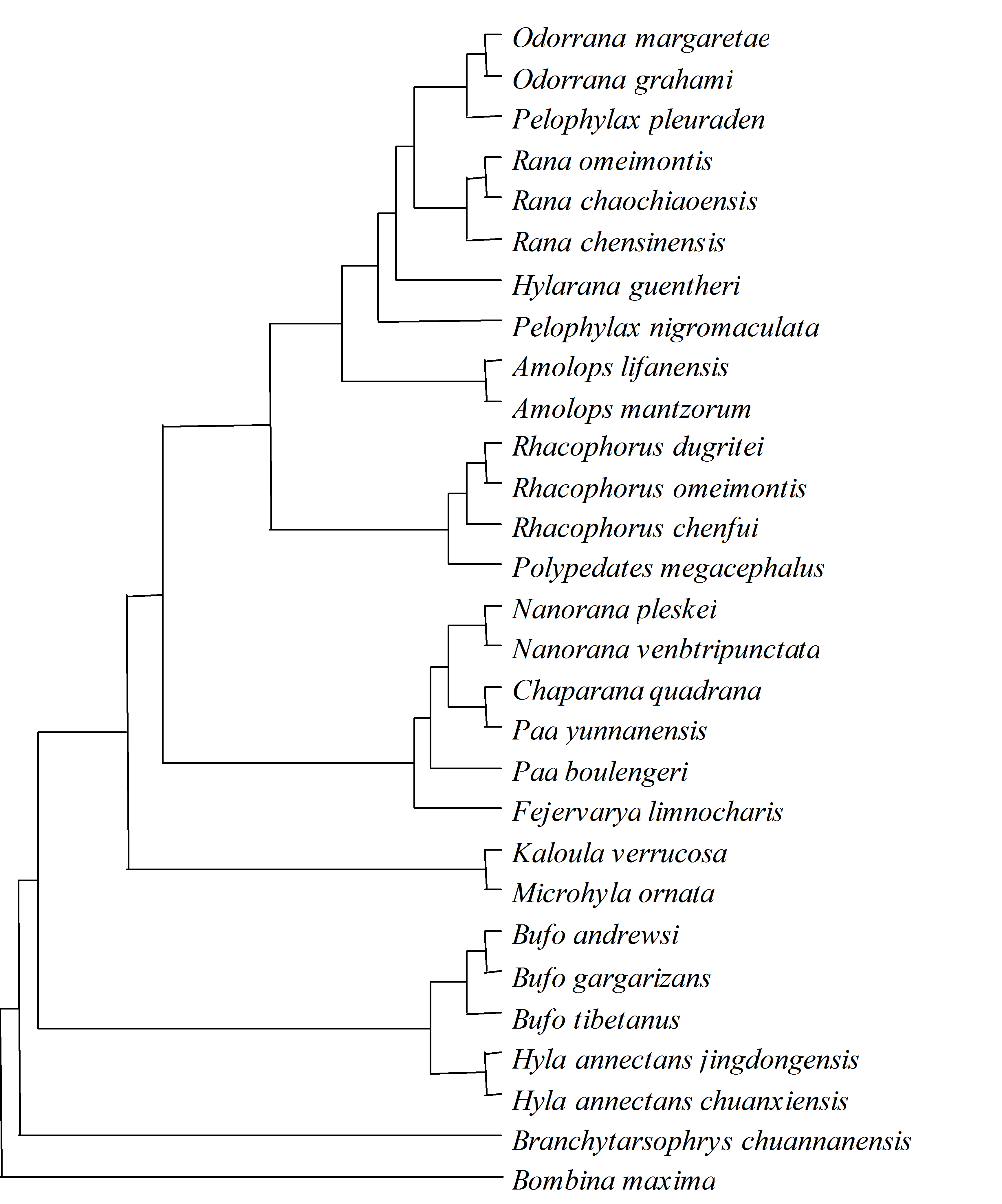

Supplement: Additional file 3: Figure S2 — The phylogenetic tree of the 29 anurans species used in the comparative analysis following Jiang et al. [51] and Pyron and Wiens [52]. [file 1471-2148-14-104-S3.jpeg]
